# Study on Welding Characteristics and Parameters of Gas Metal Arc Welding for A516 Grade 70 Steel with ER70S-6 and ER308LSi Filler Materials

**DOI:** 10.3390/ma17215391

**Published:** 2024-11-04

**Authors:** Kahwai Chong, Ervina Efzan Mhd Noor, Amalina Amir, Mirza Farrukh Baig

**Affiliations:** 1 Centre for Manufacturing and Environmental Sustainability (CMES), Faculty of Engineering and Technology, Multimedia University, Bukit Beruang, Malacca 75450, Malacca, Malaysia; 2 School of Mechanical Engineering, College of Engineering, Universiti Teknologi MARA, Shah Alam 40450, Selangor, Malaysia

**Keywords:** gas metal arc welding, filler materials, wire feeder speed, shielding gas flow rate

## Abstract

Welding is a crucial process in joining metals, especially in the fabrication industry. Thisresearch aimed to investigate the effects of using two different filler materials, ER70S-6 and ER308LSi, with nine combinations of wire feeder speed (WFS) and shielding gas flow rate (GFR), on weld joints. The study focused on the weld quality and material properties of Gas Metal Arc Welded (GMAW) butt joints of ASTM A516 G70 plates, characterized through visual inspection, liquid penetrant testing, tensile testing, hardness testing, and optical microscopy. Results indicated that the highest ultimate tensile strength and hardness were achieved at 4 m/min WFS and 15 L/min GFR with ER70S-6, and 5 m/min WFS and 20 L/min GFR with ER308LSi. The specimens welded with ER308LSi demonstrated superior mechanical properties compared to those welded with ER70S-6. Additionally, the study revealed the influence of microstructural changes from the base metal (BM) to the heat-affected zone (HAZ) and fusion zone (FZ), with finer and more compact grain structures contributing to higher hardness values. These findings underscore the importance of selecting appropriate filler materials, WFS, and GFR to achieve the desired weld quality and material properties for A516 G70 low-carbon steel welded joints.

## 1. Introduction

Welding is the fabrication process of joining two metals together by using adhesive and cohesive forces between the metals. It synthesizes the study of heat produced, melt flow solidification, and material behavior under heating and cooling cycles. In the 20th century, World War I and II accelerated the development of welding technology including safety precautions like the prevention of toxic gases and excessive UV radiation exposure [1]. Thereafter, it gave rise to welding processes such as Submerged Arc Welding (SAW), Tungsten Inert Gas (TIG), Metal Inert Gas (MIG), Electron Beam (EB) welding, and laser welding [2].

Filler material refers to a filler rod that possesses an identical composition to the base metal, filling the gap between joints to form different fillet shapes like round and oval [3]. It has a significant influence on the mechanical properties of weldment [4]. Premature failures close to the fusion zone (FZ) may result from a significant expansion mismatch between the filler material and base metal (BM) [5]. Prior research has investigated the effect of using different filler materials on welding mechanical characteristics. Satputaley et al. found the welding strength of Chromoly 4130 with filler ER70S-2 using Tungsten Inert Gas (TIG) is stronger than the weld strength of filler ER5183-welded Al7075-T6. It performs well in weld penetration without compromising material properties [6]. Ridha et al. used the filler ER70S-3 with pure argon gas to weld low-carbon steel AISI 1020 at constant voltage with different DC currents using TIG welding. In advance of welding, some of the sheet metals were shot-peened using a 1.25 mm diameter steel ball for half an hour. These metal sheets were welded in the same condition after that. It was found that the tensile strength of TIG-welded joints was 12% higher than BM [7]. Saurabh et al. concluded the welded region with filler ER70S-6 is harder than HAZ and BM, AISI 1040 [8]. Khan and Pathak used filler ER309L and ER430 of 1.2 mm diameter to pulse-MIG weld 3 mm thick cold rolled FSS430 plate. Austenitic stainless steel and ER309L filler exhibited a higher tensile strength than the ferritic stainless steel and ER430 filler. The tensile strength of each welded joint was discovered to be lower than BM due to alteration in heat treatment conditions and induced thermal stresses [9]. Shanmugasundar et al. emphasized the role of the selection of filler material by using 430 FSS plates as the BM to study the mechanical properties of filler ER310 and ER410. The tensile strength of ER410 was found to be higher than ER310 [4]. Shen et al. found the addition of filler ER410-NiMo to the base material CoCrFeMnNi using GMAW increased the cross-sectional area of FZ, leading to a lower stress per unit area in BM and HAZ under the same tensile external load. Thus, the mechanical behavior is better than the GTAW joint without filler material [10]. Shen et al. conducted similar research using CoCrFeMnNi as the base material to examine the mechanical properties of ER308LSi stainless steel filler metal. The ER308LSi filler metal increased the solidification temperature range and decreased the tensile strength at the joint compared to previous research [11]. Kellai et al. examined the PWHT to 304 austenitic stainless steel in the form of a pipe with filler ER308Si. It was found that PWHT temperature has little impact on the tensile strength of the weldment. However, it increases the ductility of the weldment at 650 °C. In addition, it was found that the weldment solution treated at 1050 °C had the most stable and best micro-hardness profile [12]. Naing and Muangjunburee suggested using ER5356 filler over ER4043 filler to repair 6082-T6 aluminum alloy joints because of the decreasing tensile and yield strength of welded joints with ER4043 filler [13]. Batahgy et al. found that ENi11 was more feasible in terms of tensile strength to match the 9% Ni steel using SMAW [14]. Asim et al. performed mechanical testing on filler Ti-5Al-2.5Sn, Ti-6Al-4V and autogenous weldment. It was found that the weldment with fillers depicted higher tensile strength than autogenous weldment and BM [15].

Various studies have optimized welding parameters to enhance the mechanical properties of welded joints using different base metals and fillers. Gandhe et al. optimized MIG arc current, voltage, and wire feed rate for low-carbon steel (AISI 1040) with ER70S-6 filler, finding that a 150 A current, 24 V voltage, and 3 m/min wire feed rate yielded higher weld strength [8]. Edy et al. demonstrated that increasing wire feeder speed and gas flow rate directly increased the hardness of SS316 metal welded using CO_2_ gas, with maximum hardness achieved at a wire feeder speed of 5 m/min and gas flow rate of 12 L/min [16]. Majeed et al. found that maximum tensile strength and hardness for AISI 1040 were attained at specific combinations of wire feed speed, welding speed, and current, noting that travel speed inversely affected tensile strength and hardness [17]. Khrais et al. optimized parameters for bead geometry and material distortion in SS316L using MAG welding, identifying specific arc current, filler feeder rate, and gas composition for optimal bead height, width, and minimal distortion [18]. Lastly, Partikno et al. explored the impact of shielding gas flow rate and V-groove type on the tensile strength of A53 steel welded with A36 steel, concluding that a double V-groove with a 25 L/min gas flow rate achieved the highest tensile strength and narrowest HAZ width [19]. These studies collectively highlight the critical influence of welding parameters on the mechanical performance and microstructure of welded joints across various metal types and welding methods.

This research aims to investigate the welding characteristics of Gas Metal Arc Welding (GMAW) for A516 Grade 70 steel using wire feeder speeds (WFSs) of 3 m/min, 4 m/min, and 5 m/min, and gas flow rates (GFRs) of 15 L/min, 20 L/min, and 25 L/min, with ER70S-6 and ER308LSi filler materials. The study compares two distinct filler materials—ER70S-6 (carbon steel) and ER308LSi (stainless steel)—to evaluate how differences in their composition affect the mechanical properties and weld quality of A516 Grade 70 low-carbon steel. While these materials differ significantly, their comparison is essential for understanding the impact of alloying elements like chromium and nickel (in stainless steel) on tensile strength, hardness, and overall weld performance. Although A516 G70 steel is widely used in pressure vessel fabrication, there is limited research on the specific effects of these welding parameters on its performance. The selected parameters are based on preliminary experiments and industry practices known to influence weld quality, mechanical properties, and microstructural characteristics. The use of stainless steel filler ER308LSi, despite its higher cost and potential challenges, is intended to explore its benefits in enhancing corrosion resistance, mechanical properties, and overall weld performance. By systematically studying these variables and filler materials, this research aims to enhance the performance and reliability of A516 G70 steel weldments, particularly in pressure vessel applications. This study provides new insights into how different fillers influence weld performance and offers valuable data for optimizing welding parameters, helping guide material selection and parameter optimization for industries requiring high-quality, reliable welds in critical applications.

## 2. Materials and Methods

### 2.1. Materials and Welding

In this study, ASTM A516 Grade 70 was selected as the base metal because it is a commercially available low-carbon steel commonly used in low-temperature pressure vessels due to its excellent weldability and formability. Its low carbon content helps minimize the risk of cracking during welding, ensuring more reliable and consistent results. The A516 Grade 70 plate was machined into dimensions of 120 mm × 120 mm × 10 mm (length × width × thickness) with a 60° V-shaped groove, a 1.5 mm root face, and a 2 mm root gap. GMAW was performed using 0.8 mm diameter ER70S-6 and ER308LSi fillers with DCEP polarity and a constant voltage of 18 V. Information on the base metal was retrieved from ASTM [20], and the welding wires were obtained from the supplier, Leeden Powerweld Sdn. Bhd [21]. Using a 0.8 mm diameter welding wire for a 10 mm thick plate in GMAW allows for precise control over heat input, penetration, and deposition rate, ensuring high-quality welds with good mechanical properties. The groove design and welding parameters further enhance the effectiveness of this approach, making it a practical choice for welding thicker plates. The chemical compositions and mechanical properties of the base metal and filler materials are provided in Table 1 and Table 2. The welding process parameters are tabulated in Table 3 and Table 4.

### 2.2. Testing and Evaluation Methods

The quality and mechanical properties of the welds were evaluated using a combination of non-destructive testing (NDT), metallographic analysis, and mechanical testing. Visual inspection was first conducted to identify surface imperfections, ensuring proper cleaning and lighting for optimal detection. Following this, the Liquid Penetrant Test (LPT) was applied to detect surface cracks and discontinuities, especially useful for non-ferromagnetic materials like stainless steel.

For metallographic analysis, specimens were prepared by progressively polishing them with abrasive papers of various grit sizes (240 to 1000), followed by a final polish with a 0.05 μm alumina slurry. The ER70S-6 weld joint was etched with Nital solution, while the ER308LSi joint was etched with a mixture of hydrochloric acid and ferric chloride. Cross-sectional samples were taken perpendicular to the welding path to capture the base metal (BM), heat-affected zone (HAZ), and fusion zone (FZ).

Mechanical testing included hardness measurements at the FZ, HAZ, and BM using a 2 kg load for 10 s, with three test points for each zone. Tensile test specimens were prepared according to ASTM E8 standards [22], with dimensions of 100 mm gauge length, 20 mm width, and 6 mm thickness. These were cut transversely along the welding path and milled to a uniform thickness of 6 mm. The tensile tests were performed with a crosshead speed of 0.05 mm/min to determine the mechanical properties of the weld joints.

## 3. Results

### 3.1. Visual Inspection and Liquid Penetrant Test

**Figure 1:**
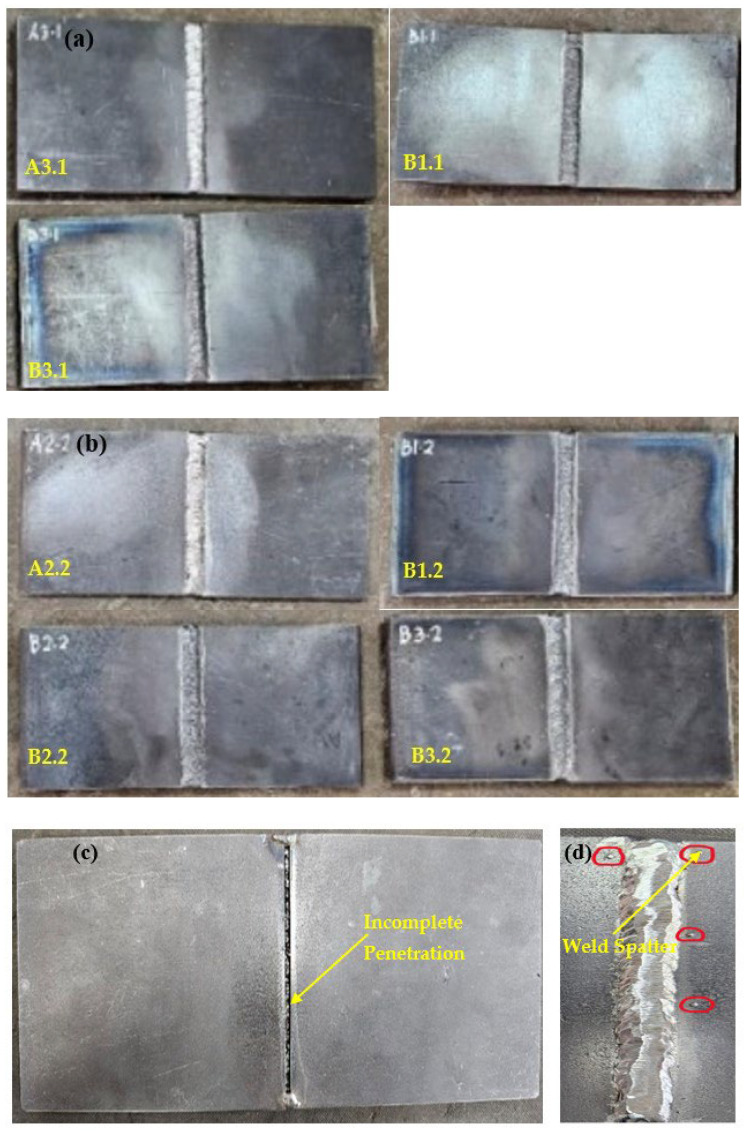
(**a**) Specimens A3.1, B1.1, and B3.1 (**b**) Specimens A2.2, B1.2, B2.2, and B3.2 (**c**) Incomplete penetration (specimen A3.1) (**d**) Weld spatter (Specimen A2.2).

Figure 1 presents an analysis of welding defects identified through visual inspection and LPT. In Figure 1a, the front sides of specimens A3.1, B1.1, and B3.1 demonstrate a consistent weld bead with no obvious visual defects, indicating a good quality weld. The surface appears clean, and the uniformity of the weld bead suggests proper welding technique. Figure 1b displays the front side of specimens A2.2, B1.2, B2.2, and B3.2, showing a consistent and uniform weld bead with no major visible defects. Figure 1c highlights defects observed on the backside of the specimen A3.1 from Figure 1a, which was welded with a wire feeder speed (WFS) of 3 m/min and a gas flow rate (GFR) of 25 L/min. It shows incomplete penetration, where the weld does not fully penetrate the joint thickness, potentially compromising the weld’s strength and integrity. Meanwhile, Figure 1d illustrates weld spatter along the welding path of the specimen A2.2 from Figure 1b, with a WFS of 4 m/min and a GFR of 20 L/min. Weld spatter consists of small droplets of molten material scattered around the weld area. The rest of the specimens are defect-free.

### 3.2. Microstructural Evaluation

**Figure 2:**
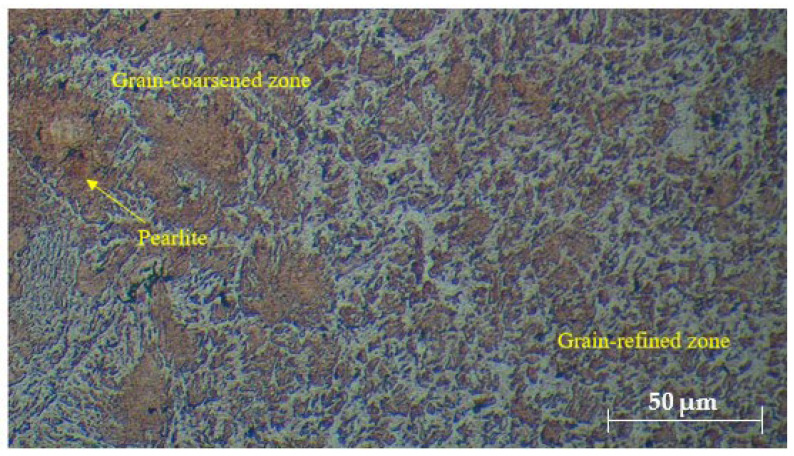
Grain structures in the upper part of HAZ.

**Figure 3:**
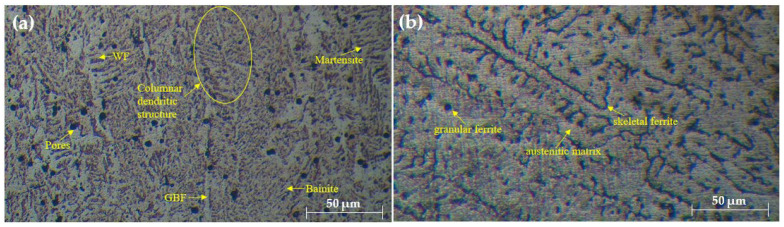
Microstructure of FZ welded with (**a**) ER70S-6 (**b**) ER308LSi filler.

**Figure 4:**
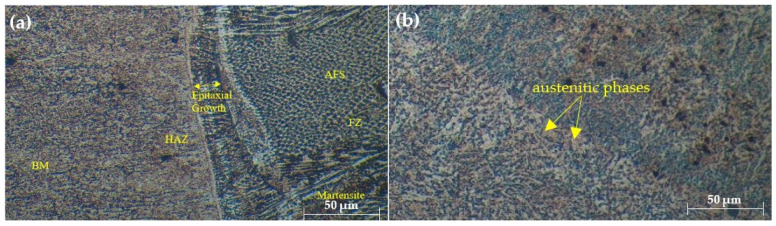
Microstructure of HAZ welded with (**a**) ER70S-6 (**b**) ER308LSi filler.

The microstructural evaluation of the welded joints, as shown in Figure 2, Figure 3 and Figure 4, provides insightful details about the grain structures and microstructural features resulting from different filler materials. Figure 2 reveals the grain structures in the upper part of the HAZ, distinguishing the grain-coarsened zone with larger grains due to high welding temperatures, the pearlite region indicating slower cooling, and the grain-refined zone with smaller grains formed through recrystallization. In Figure 3, the FZ microstructure is depicted for welds using ER70S-6 and ER308LSi fillers. The ER70S-6 filler shows a columnar dendritic structure, martensite, bainite, and some porosity, with a some small number of specific morphologies of ferrite such as Widemänstatten ferrite (WF) and grain boundary ferrite (GBF) also found in the FZ, aligning with the microstructure results obtained by Khamari et al. [23]. In contrast, the ER308LSi filler exhibits granular ferrite, skeletal ferrite, and an austenite matrix, each phase contributing to the weld’s mechanical properties. Two morphological structures, granular ferrite and skeletal ferrite, are seen to be equally distributed all over the center of the FZ. These structures are categorized under types of δ-ferrite. δ-ferrite can be brought to room temperature after a cooling process due to the high percentage of nickel and chromium content in the ER308LSi filler [24]. Figure 4 focuses on the HAZ microstructure, highlighting features such as epitaxial growth and acicular ferrite structure (AFS) in welds with the ER70S-6 filler and typical austenitic phases with the ER308LSi filler. These micrographs collectively demonstrate the distinct microstructural characteristics imparted by different welding conditions and filler materials, underscoring their influence on the welded joints’ overall properties.

### 3.3. Mechanical Testing

#### 3.3.1. Tensile Test

Tensile testing was conducted in the uniaxial direction to the welding path, and the results are presented in Table 5. The highest ultimate tensile strength (UTS) obtained using ER70S-6 and ER308LSi fillers were 302.7 MPa for specimen A1.2 and 444.9 MPa for specimen B2.3, respectively. This significant difference in UTS highlights the superior mechanical properties imparted by the ER308LSi filler compared to ER70S-6, likely due to the higher chromium and nickel content in ER308LSi, which enhances its strength and ductility.

**Figure 5:**
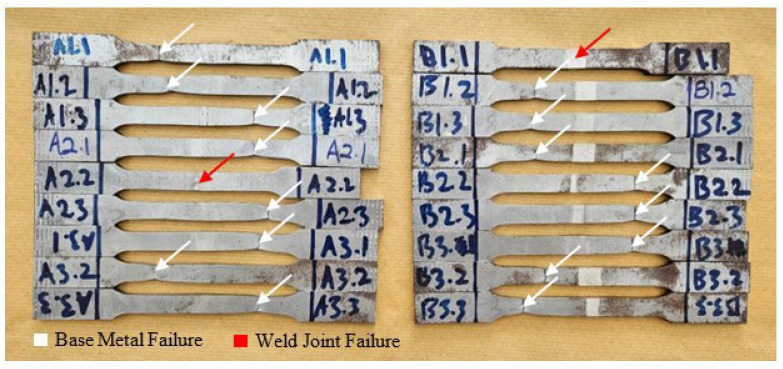
Failure location of the tensile specimens.

**Figure 6:**
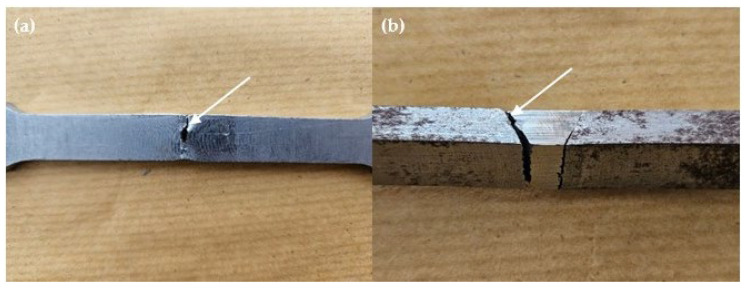
Fractured location on weld joints of specimens (**a**) A2.2 (**b**) B1.1.

The fracture location in Figure 5 indicates the weakest part of the tensile specimen. In this case, the base metal is identified as the weakest part because most specimens fractured at the base metal, except for specimens A2.2 and B1.1. In Figure 6a, the fracture behavior observed in specimen A2.2, where failure occurred in the FZ rather than the base metal, is hypothesized to be due to the formation of martensitic or bainitic phases. Although specimens with higher wire feeder speeds (WFSs) and gas flow rates (GFRs) were also tested, the specific combination of a 4 m/min WFS and a 20 L/min GFR in specimen A2.2 may have resulted in localized cooling conditions that favored the formation of harder, more brittle microstructures. Martensite and bainite formation occurs during rapid cooling, where austenite transforms into these harder phases. The FZ in specimen A2.2 may have experienced localized rapid cooling due to slight variations in heat dissipation, joint geometry, or thermal cycling, even though the overall heat input was not the highest compared to other specimens. These microstructural transformations can lead to increased hardness but reduced ductility, making the weld more prone to fracture under tensile stress. While direct evidence of martensite or bainite formation was not confirmed through microstructural analysis, the increased hardness and the brittle fracture observed in the FZ of specimen A2.2 suggest that such phases may have formed. This assumption is based on the well-documented metallurgical behavior of low-carbon steels under similar cooling conditions, where rapid cooling tends to produce martensitic or bainitic structures. Therefore, the fracture in the FZ of specimen A2.2 is attributed to the likely presence of these brittle phases, whereas other specimens, with different welding parameters, fractured in the base metal where the microstructure remained more ductile.

Specimen B1.1 exhibited lower tensile strength compared to the other specimens, which can be attributed to insufficient fusion between the filler material and the base metal, as indicated by the microstructural analysis in Figure 6b. The lack of complete sidewall fusion suggests that the welding parameters, particularly the heat input, may not have been sufficient to achieve full penetration and bonding in the weld joint. Incomplete sidewall fusion can occur when the heat input is not high enough to fully melt the base metal and integrate the filler material across the entire weld interface. This observation underscores the importance of optimizing welding parameters such as WFS and GFR to ensure proper fusion and uniform distribution of heat during the welding process. The reduced tensile strength in specimen B1.1 highlights the sensitivity of weld quality to these parameters.

Moreover, the observed differences in tensile strength and fracture locations between specimens welded with ER70S-6 and ER308LSi fillers underscore the importance of filler material selection in welding applications. The chemical composition of the filler material plays a crucial role in determining the overall mechanical properties of the welded joint. The stainless steel filler ER308LSi, with its higher content of alloying elements such as chromium and nickel, provides better tensile strength and ductility compared to the carbon steel filler ER70S-6.

The highest UTS obtained for the ER70S-6 filler occurred at a wire feeder speed (WFS) of 4 m/min and a gas flow rate (GFR) of 15 L/min. Similarly, the highest UTS for the ER308LSi filler was achieved at a WFS of 5 m/min and a GFR of 20 L/min. Despite these specific conditions yielding the highest UTS values, no significant trend in UTS was observed with changes in WFS and GFR across the specimens.

This analysis reveals that while optimal parameters for maximum UTS can be identified, the overall UTS does not consistently increase or decrease with variations in WFS or GFR. This suggests that other factors, such as heat input, arc stability, and filler metal composition, also play crucial roles in determining the tensile strength of the welded joints. For instance, the higher UTS observed with the ER308LSi filler could be attributed to its enhanced alloy composition, including higher chromium and nickel content, which contributes to improved strength and ductility.

#### 3.3.2. Hardness Test

**Figure 7:**
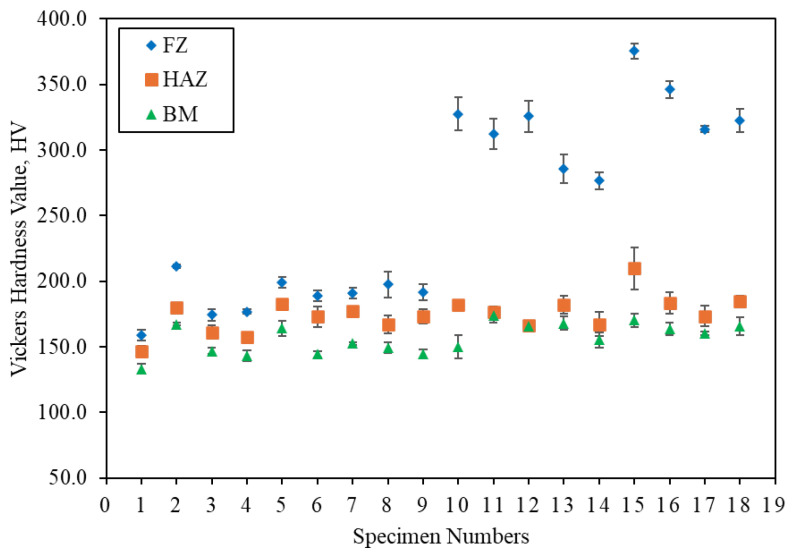
Vickers hardness at various zones, for ER70S-6 (specimens 1–9), and ER308LSi (specimens 10–18).

Figure 7 shows the graph of Vickers hardness at the FZ, HAZ, and BM for all the specimens. Specimens numbered 1 to 9 correspond to those labeled A1.1 to A3.3, and specimens numbered 10 to 18 correspond to those labeled B1.1 to B3.3. A consistent trend is observed among the FZ, HAZ, and BM, where the hardness of the FZ is higher than that of the HAZ, and the hardness of the HAZ is higher than that of the BM, as shown in Figure 7. This observation aligns with the findings of Gandhe et al. [8]. According to research by Opiela et al., fine grains in the microstructure result in higher hardness values compared to coarse grains [25]. In the microstructural evaluation section, it was mentioned that the grain size becomes more refined and compact moving from the center cross-section of the BM to the direction of the HAZ and FZ. Thus, the results are validated by the obtained hardness values.

Moreover, the higher hardness of the FZ compared to the HAZ and BM in the specimens labeled A is due to the presence of localized martensite and bainite. In comparing carbon steel filler (ER70S-6) and stainless steel filler (ER308LSi), the FZ hardness of the stainless steel filler is higher than that of the carbon steel filler, which is attributed to the presence of δ-ferrite [4,24]. Similarly, the results obtained in the tensile test indicate that a combination of a wire feeder speed of 4 m/min and a gas flow rate of 15 L/min achieves the highest hardness among specimens using the ER70S-6 filler, while a combination of 5 m/min and 20 L/min achieves the highest hardness among specimens using the ER308LSi filler. This correlation suggests that the results obtained for both the tensile test and Vickers hardness test are accurate, as materials with high UTS tend to have high hardness values.

## 4. Conclusions

Based on the conducted tests, the following conclusions can be made.

1. Using the ER70S-6 carbon steel filler, the highest ultimate tensile strength (UTS) of 302.7 MPa and a hardness value of 211.3 HV are achieved with a combination of a 4 m/min wire feeder speed and a 15 L/min shielding gas flow rate. This result can be attributed to the optimal balance between heat input and cooling rate provided by this specific combination of wire feeder speed and gas flow rate. The moderate heat input allows for good penetration and fusion without overheating the weld, reducing the risk of defects such as excessive grain growth or incomplete fusion. Additionally, the controlled cooling rate prevents the formation of excessive martensite in the fusion zone, leading to a reasonable hardness value, while ensuring a ductile weld with adequate tensile strength.
2. Welding with the ER308LSi stainless steel filler, the highest UTS of 444.9 MPa and a hardness value of 375.4 HV are achieved with a combination of a 5 m/min wire feeder speed and a 20 L/min shielding gas flow rate. The higher UTS and hardness in this case are due to the alloying elements in ER308LSi, particularly chromium and nickel, which enhance the weld’s mechanical properties. The increased wire feeder speed introduces more filler material into the weld pool, resulting in a denser and stronger weld. Meanwhile, the 20 L/min gas flow rate provides optimal shielding, preventing oxidation and ensuring a clean, high-quality weld. The formation of δ-ferrite in the fusion zone contributes to the increased hardness, although care must be taken as high hardness can also increase brittleness.
3. The grain structure changes from the base metal (BM) to the heat-affected zone (HAZ) and from the HAZ to the fusion zone (FZ). The grain size becomes finer and more compact as it moves from the BM to the FZ. This observation is validated by the hardness results, which show that the FZ has a higher hardness than the HAZ, and the HAZ has a higher hardness than the BM. The presence of fine grains in the microstructure contributes to higher hardness values. These conditions are valid under the observed microstructural characteristics, where recrystallization and thermal cycling result in grain refinement.
4. The higher hardness of the FZ compared to the HAZ and BM is attributed to the presence of localized martensite and bainite. Additionally, the FZ hardness of the stainless steel filler ER308LSi is higher than that of the carbon steel filler ER70S-6 due to the formation of δ-ferrite. However, high hardness can increase brittleness, making the material prone to cracking at low temperatures. This condition is valid in scenarios where the thermal history of the welding process promotes the formation of martensitic and bainitic structures and the chemical composition of the filler material influences the formation of δ-ferrite. Incomplete sidewall fusion may also occur due to the different chemical compositions between the stainless steel weldment and the carbon steel base metal, leading to lower mechanical integrity in certain weld conditions.
5. Therefore, for welding ASTM A516 Grade 70 low-carbon steel, the ER70S-6 filler with a 4 m/min wire feeder speed and a 15 L/min shielding gas flow rate is the preferred choice. This combination provides optimal mechanical properties while minimizing the risk of brittleness and ensuring better fusion.

## Figures and Tables

**Table 1:** Chemical compositions of A516 Grade 70, ER70S-6 and ER308LSi.

Materials	C	Mn	Si	P	S	Cr	Ni	Fe
A516 G70	0.27	0.79–1.30	0.13–0.45	0.035max.	0.035max.	-	-	Balance
ER70S-6	0.1	1.45	0.88	0.012	0.014	-	-	Balance
ER308LSi	0.025	2.25	0.78	0.019	0.009	19.9	10.2	Balance

**Table 2:** Mechanical properties of A516 Grade 70, ER70S-6 and ER308LSi.

Materials	Yield Stress [MPa]	Ultimate Tensile Strength [MPa]	Elonglation [%EL]
A516 G70	260	485–620	21
ER70S-6	460	560	28
ER308LSi	-	590	42

**Table 3:** Welding process parameters for filler ER70S-6.

Filler ER70S-6				
	Gas Flow Rate (L/min)	15	20	25
Wire Feeder Speed (m/min)				
3		A1.1	A2.1	A3.1
4		A1.2	A2.2	A3.2
5		A1.3	A2.3	A3.3

**Table 4:** Welding process parameters for filler ER308LSi.

Filler ER308LSi				
	Gas Flow Rate (L/min)	15	20	25
Wire Feeder Speed (m/min)				
3		B1.1	B2.1	B3.1
4		B1.2	B2.2	B3.2
5		B1.3	B2.3	B3.3

**Table 5:** Ultimate tensile strength of different specimens made from A516 Grade 70 with fillers ER70S-6 and ER308LSi.

Specimen	Wire Feeder Speed (m/min)	Gas Flow Rate (L/min)	Ultimate Tensile Strength (MPa)	Failure
A1.1	3	15	300.5	Base Metal
A1.2	4	15	302.7	Base Metal
A1.3	5	15	292.0	Base Metal
A2.1	3	20	287.4	Base Metal
A2.2	4	20	281.9	Weld Joint
A2.3	5	20	291.0	Base Metal
A3.1	3	25	292.8	Base Metal
A3.2	4	25	292.4	Base Metal
A3.3	5	25	294.8	Base Metal
B1.1	3	15	194.5	Weld Joint
B1.2	4	15	311.6	Base Metal
B1.3	5	15	307.3	Base Metal
B2.1	3	20	299.6	Base Metal
B2.2	4	20	287.9	Base Metal
B2.3	5	20	444.9	Base Metal
B3.1	3	25	301.3	Base Metal
B3.2	4	25	320.9	Base Metal
B3.3	5	25	300.2	Base Metal

## Data Availability

The data presented in this study are available in the article.
